# EQ-5D full health state after therapy heralds reduced hazard to accrue subsequent organ damage in systemic lupus erythematosus

**DOI:** 10.3389/fmed.2022.1092325

**Published:** 2022-12-20

**Authors:** Julius Lindblom, Sture Zetterberg, Sharzad Emamikia, Alexander Borg, Gunilla von Perner, Yvonne Enman, Emelie Heintz, Malin Regardt, David Grannas, Alvaro Gomez, Ioannis Parodis

**Affiliations:** ^1^Division of Rheumatology, Department of Medicine Solna, Karolinska Institutet, Karolinska University Hospital, Stockholm, Sweden; ^2^Swedish Rheumatism Association, Stockholm, Sweden; ^3^Department of Learning, Informatics, Management and Ethics (LIME), Karolinska Institutet, Stockholm, Sweden; ^4^Department of Neurobiology, Care Sciences and Society, Karolinska Institutet, Stockholm, Sweden; ^5^Medical Unit Occupational Therapy and Physiotherapy, Karolinska University Hospital, Stockholm, Sweden; ^6^Division of Biostatistics, Institute of Environmental Medicine, Karolinska Institutet, Stockholm, Sweden; ^7^Department of Rheumatology, Faculty of Medicine and Health, Örebro University, Örebro, Sweden

**Keywords:** systemic lupus erythematosus, organ damage, patient-reported outcomes, patient perspective, outcomes research, health-related quality of life

## Abstract

**Objectives:**

To investigate whether self-reported EQ-5D full health state (FHS) after therapeutic intervention for active systemic lupus erythematosus (SLE) is associated with a reduced risk to accrue organ damage. In a separate analysis, we sought to investigate associations between experience of “no problems” in each one of the five dimensions of EQ-5D and the risk to accrue damage.

**Methods:**

Data from the open-label extension periods of the BLISS-52 and BLISS-76 trials of belimumab in SLE (NCT00724867; NCT00712933) were used (*N* = 973). FHS was defined as an experience of “no problems” in all five EQ-5D dimensions. Organ damage was assessed annually using the Systemic Lupus International Collaborating Clinics (SLICC)/American College of Rheumatology (ACR) Damage Index (SDI). Associations between the three-level version of the EQ-5D (EQ-5D-3L) responses at open-label baseline and the first documented increase in organ damage were investigated using Cox regression accounting for age, sex, ancestry, SDI at baseline, and background therapy, and associations with SDI items were investigated using phi (φ) correlation analyses.

**Results:**

A total of 147 patients (15.1%) accrued organ damage during follow-up, with the first increase in their SDI score occurring after a mean time of 29.1 ± 19.6 months. Lower proportions of FHS respondents accrued damage over a course of up to 7.9 years of open-label follow-up compared with no FHS respondents (*p* = 0.004; derived from the logrank test). FHS was associated with a reduced hazard to accrue subsequent organ damage (HR: 0.60; 95% CI: 0.38–0.96; *p* = 0.033) after adjustments, as was experience of “no problems” in mobility (HR: 0.61; 95% CI: 0.43–0.87; *p* = 0.006). “No problems” in mobility was negatively correlated with musculoskeletal damage accrual (φ = −0.08; *p* = 0.008) and associated with a lower hazard to accrue musculoskeletal damage in Cox regression analysis (HR: 0.38; 95% CI: 0.19–0.76; *p* = 0.006).

**Conclusion:**

Experience of EQ-5D-3L FHS and “no problems” in mobility after therapeutic intervention heralded reduced hazard to accrue subsequent organ damage, especially musculoskeletal damage, suggesting that optimisation of these health-related quality of life aspects constitutes a clinically relevant treatment target in patients with SLE, along with clinical and laboratory parameters.

## 1 Introduction

Systemic lupus erythematosus (SLE) is a chronic autoimmune disease that is characterised by considerable morbidity and impaired health-related quality of life (HRQoL) (1, 2). Organ damage is a particularly feared and irreversible consequence of the disease; approximately 50% of SLE patients accrue damage to some extent within 10 years of receiving their diagnosis, including damage in the musculoskeletal system, central nervous system (CNS), and kidneys (3). SLE is a highly heterogeneous disease (4); afflicted individuals differ not only in clinical presentation but also in how they experience their health, commonly experiencing a poor HRQoL, even after adequate clinical response to therapy (5–7).

During the Outcome Measures in Rheumatoid Arthritis Clinical Trials (OMERACT) IV consensus conference, four principal outcome domains were ratified for SLE clinical trials: (i) disease activity, (ii) HRQoL, (iii) adverse events, and (iv) organ damage (8). Because of the known discordance between SLE patients and physicians regarding concerns for disease features as well as perception of disease activity (9), optimal use of patient-reported outcomes is highly motivated both in clinical trial and real-life settings (10).

The three-level version of EQ-5D (EQ-5D-3L) is a patient-reported HRQoL measure that includes a questionnaire with five dimensions of health (11, 12). Each one of these dimensions yield patient responses at three severity levels i.e., level 1, 2, and 3, for experience of “no problems,” “some/moderate problems,” and “extreme/major problems,” respectively. Combinations of patient responses in this descriptive system represent the patient’s self-reported health status, which is referred to as an EQ-5D profile (12). These profile data can be supplemented by a weighting system which converts the EQ-5D profile into a single value, referred to as an EQ-5D index score, ranging from lower than 0 to 1, where 0 represents health state experience equal to death and 1 represents a health state equivalent to full health, also denoted as full health state (FHS) (12).

The relationship between health status reported using EQ-5D and SLE disease activity or organ damage was investigated in a meta-analysis by Shi et al. (13) which however, yielded inconclusive results. Aggarwal et al. (14) showed that EQ-5D was able to discriminate between patient subgroups of high and low disease activity but did not manage to differentiate between patient subgroups of high and low organ damage. Wang et al. (15) demonstrated a negative association between EQ-5D values and organ damage while a study by Chang et al. (16) showed a weaker association. The association between EQ-5D scores and organ damage in patients with SLE is thus not clear and warrants further research.

Three-level version of EQ-5D FHS was reported at a higher frequency among patients who received non-biological standard therapy plus belimumab than among patients who received standard therapy alone as well as in responders than in non-responders in a large clinical trial SLE population (17). However, the association between EQ-5D-3L FHS and long-term outcomes e.g., organ damage accrual in SLE remains unknown.

The aim of this study was to investigate whether experience of EQ-5D-3L FHS after therapy for active SLE is associated with deceleration of organ damage accrual in a diverse SLE population deriving from open-label studies of belimumab. In a separate analysis, we sought to investigate associations between experience of “no problems” in each one of the five dimensions of EQ-5D and the risk to accrue damage.

## 2 Materials and methods

### 2.1 Study design and population

This study was conducted as a *post hoc* analysis of data collected in the setting of the BLISS-52 (NCT00424476) (18) and BLISS-76 (NCT00410384) (19) trials, and their open-label extension phases (NCT00724867; NCT00712933) (20, 21). A total of 973 participants with open-label extension phase data were included in the present study, comprising 528 and 445 participants from the BLISS-52 and BLISS-76 trials, respectively. Access to data was granted by GlaxoSmithKline (Uxbridge, UK) through the Clinical Study Data Request (CSDR) consortium.

All patients included in the BLISS-52 and BLISS-76 trials fulfilled the American College of Rheumatology (ACR) revised criteria for SLE (22), were adults, had an ANA titre ≥1:80 and/or serum antibodies against double-stranded DNA (anti-dsDNA) antibody level ≥30 IU/mL at screening, and a Safety of Estrogens in Lupus National Assessment-SLEDAI (SELENA-SLEDAI) score ≥6 (23). All patients were on stable non-biological standard therapy for ≥30 days before the baseline of the double-blinded phases of the BLISS-52 and BLISS 76 trials, including glucocorticoids, antimalarial agents, and/or immunosuppressants. Patients were randomised to receive add-on belimumab 1 mg/kg, belimumab 10 mg/kg, or placebo as intravenous infusions at weeks 0, 2, 4, and thereafter every 4th week. Participants from the BLISS-52 and BLISS-76 clinical trials all received belimumab 10 mg/kg intravenously every 4th week on top of non-biological standard therapy during the open-label phase of the studies and formed the study population of the present investigation.

### 2.2 EQ-5D-3L full health state and dimensions

The EQ-5D-3L descriptive system incorporates five HRQoL dimensions i.e., Mobility, Self-care, Usual activities, Pain/discomfort, and Anxiety/depression. Respondents may report no problems, some/moderate, or extreme/major problems in each one of these dimensions. As per the EQ-5D-3L user guide, we defined FHS as a response of “no problems” in all five dimensions, which yields an EQ-5D-3L index score of 1 (12) at the open-label baseline, which corresponded to the end of follow-up in the BLISS-52 trial at week 52 and the BLISS-76 trial at week 76. In subgroup analyses, we also analysed patient responses of “no problems” in each one of the five EQ-5D dimensions separately.

### 2.3 Clinical definitions

Systemic lupus erythematosus disease activity was assessed using the SLEDAI 2000 (SLEDAI-2K) (24) and organ damage was assessed using the Systemic Lupus International Collaborating Clinics (SLICC)/ACR Damage Index (SDI) (25). The SDI includes 39 items grouped into 12 domains i.e., ocular, neuropsychiatry, renal, pulmonary, cardiovascular, peripheral vascular, gastrointestinal, musculoskeletal, skin, premature gonadal failure, diabetes, and malignancy. Organ damage accrual was defined as the first documented increase in the patient’s SDI score from the open-label baseline over the open-label extension study period, based on yearly assessments for a total duration of up to 8 years.

### 2.4 Statistics

Proportional hazards (Cox) regression was used to investigate associations between EQ-5D-3L FHS at the open-label baseline and organ damage accrual, both overall and stratified by organ domain, during the open-label extension period. In addition, associations between experience of “no problems” in each one of the five HRQoL dimensions of EQ-5D and organ damage accrual were assessed. Kaplan–Meier survival curves were used for illustrating the time to SDI increase, and the logrank test was used for unadjusted comparisons between groups. Adjustments for possible confounders were conducted using multivariable Cox regression models; covariates in these models included age, sex, ancestry, open-label baseline degree of organ damage, and background therapy i.e., antimalarial agent and immunosuppressant use at the open-label baseline, and mean prednisone or equivalent dose during open-label follow-up. Correlations between FHS or experience of “no problems” in each one of the five EQ-5D HRQoL dimensions and damage accrual were assessed using phi (φ) correlation coefficients. This analysis included stratification into SDI domains. Data are presented in the form of numbers (percentage) or means (standard deviation) while medians (interquartile range) are indicated in the case of non-normal distributions. Comparisons of unrelated continuous data were made using the Mann–Whitney U test, and associations between unrelated binomial variables were investigated using Pearson’s chi squared (χ^2^) or Fisher’s exact tests as appropriate. All *p*-values < 0.05 were considered statistically significant. Analyses were performed using the R software version 4.1.0 (R Foundation for Statistical Computing, Vienna, Austria).

### 2.5 Ethics

The study complied with the ethical principles of the Declaration of Helsinki. Written informed consent was obtained from all study participants prior to enrolment in BLISS-52 and BLISS-76 and their open-label extension phases. The BLISS study protocols were reviewed and approved by regional ethics review boards for all participating centres, and the study protocol for this *post hoc* analysis was reviewed and approved by the Swedish Ethical Review Authority (2019-05498).

### 2.6 Patient involvement

Patient research partners were involved in the study concept and design, interpretation of data, and editing of the manuscript.

## 3 Results

Patient characteristics and clinical data at the open-label baseline, as well as comparisons between patients who experienced an increase in SDI during follow-up (*N* = 147) and patients who did not (*N* = 826) are presented in Table 1. The mean observation time for the entire open-label patient population was 49.7 ± 25.5 months. A total of 147 patients (15.1%) accrued organ damage during follow-up, with the first increase in their SDI score occurring after a mean time of 29.1 ± 19.6 months. In the group of patients who experienced an increase in SDI scores, a greater proportion had organ damage at the open-label baseline compared with their counterparts (56.5% versus 39.7%; *p* < 0.001). Among patients who reported FHS at the open-label baseline, the proportion of patients who experienced an increase in SDI scores during the subsequent follow-up was lower compared with that of patients who did not accrue damage (15.0% versus 26.6%; *p* = 0.004).

**TABLE 1:** Characteristics and comparisons between patients who displayed an increase in SDI during follow-up and patients who did not.

	All patients	SDI increase	No SDI increase	*P*-value
	*N* = 973	*N* = 147	*N* = 826	
Patient characteristics				
Age at baseline (years)	38.4 ± 11.5	41.7 ± 11.7	37.8 ± 11.3	**<0.001**
Female sex	915 (94.0%)	139 (94.6%)	776 (93.9%)	0.921
Ancestries				
Asian	209 (21.5%)	29 (19.7%)	180 (21.8%)	0.651
Black/African American	74 (7.6%)	21 (14.3%)	53 (6.4%)	**0.002**
Indigenous American*	230 (23.6%)	27 (18.4%)	203 (24.6%)	0.127
White/Caucasian	460 (47.3%)	70 (47.6%)	390 (47.2%)	0.999
Clinical data				
SLE duration at baseline (years)	4.3 (1.6–9.3)	5.5 (2.0–10.8)	4.2 (1.5–9.1)	**0.042**
SLEDAI-2K score at baseline	5.2 ± 3.6	5.9 ± 3.9	5.0 ± 3.5	**0.015**
SDI score at baseline	0.8 ± 1.2 0.0 (0.0–1.0)	1.1 ± 1.4 1.0 (0.0–2.0)	0.7 ± 1.2 0.0 (0.0–1.0)	**<0.001**
SDI score > 0 at baseline	411 (42.2%)	83 (56.5%)	328 (39.7%)	**<0.001**
EQ-5D-3L FHS at baseline	242 (24.9%)	22 (15.0%)	220 (26.6%)	**0.004**
Mean prednisone equivalent dose during follow-up (mg)	9.6 ± 8.0; *N* = 972	9.1 ± 8.2	9.7 ± 8.0; *N* = 825	0.293
Antimalarial agents use^†^	644 (66.2%)	95 (64.6%)	549 (66.5%)	0.734
Immunosuppressants use	434 (44.6%)	75 (51.0%)	359 (43.5%)	0.108
Azathioprine use	218 (22.4%)	37 (25.2%)	181 (21.9%)	0.444
Methotrexate use	114 (11.7%)	22 (15.0%)	92 (11.1%)	0.234
Mycophenolic acid use	94 (9.7%)	14 (9.5%)	80 (9.7%)	1.000
Other immunosuppressants^‡^ use	14 (1.4%)	2 (1.4%)	12 (1.5%)	1.000
Trial intervention (prior to open-label baseline)				
Placebo	309 (31.8%)	36 (24.5%)	273 (33.1%)	0.050
Belimumab 1 mg/kg	338 (34.7%)	55 (37.4%)	283 (34.3%)	0.518
Belimumab 10 mg/kg	326 (33.5%)	56 (38.1%)	270 (32.7%)	0.236

Data are presented as numbers (percentage) or means ± standard deviation. In case of non-normal distributions, the medians (interquartile range) are indicated. In case of missing values, the total numbers of patients with available data are indicated. Statistically significant p-values are in bold. EQ-5D-3L, three-level version of EQ-5D; FHS, full health state; SDI, SLICC/ACR Damage Index; SLE, systemic lupus erythematosus; SLEDAI-2K, SLE Disease Activity Index 2000.

*Alaska Native or American Indian from North, South, or Central America.

^†^Hydroxychloroquine, chloroquine, mepacrine, mepacrine hydrochloride, or quinine sulfate.

^‡^Cyclosporine, oral cyclophosphamide, leflunomide, mizoribine, or thalidomide. Statistically significant p-values are in bold.

### 3.1 Full health state experience in relation to organ damage accrual

**FIGURE 1:**
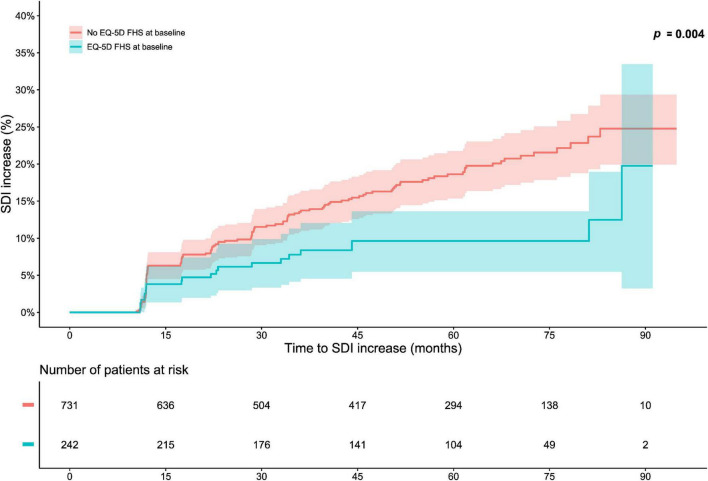
Full health state (FHS) in relation to organ damage accrual. The graphs in the upper panel delineate proportions of patients who accrued organ damage over the course of the open-label study period, stratified into patients who experienced EQ-5D-3L FHS at the open-label baseline and patients who did not. The lower panel shows numbers of participants in the two groups over time, decreasing due to documentation of SDI increase or data censoring. EQ-5D-3L, three-level version of EQ-5D; FHS, full health state; SDI, Systemic Lupus International Collaborating Clinics (SLICC)/American College of Rheumatology (ACR) Damage Index.

**FIGURE 2:**
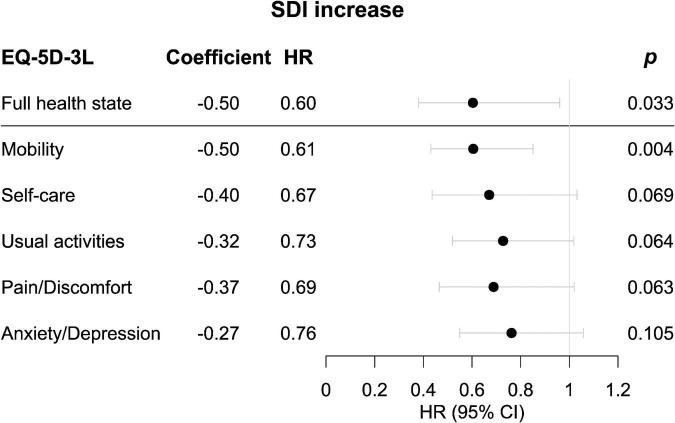
Associations of experience of full health state (FHS) or “no problems” in the separate dimensions of EQ-5D-3L in relation to organ damage accrual. Forest plots illustrating results from multivariable proportional hazards (Cox) regression analysis. Circles represent HRs and whiskers denote the 95% CI. CI, confidence interval; EQ-5D-3L, three-level version of EQ-5D; FHS, full health state; HR, hazard ratio; SDI, Systemic Lupus International Collaborating Clinics (SLICC)/American College of Rheumatology (ACR) Damage Index.

In the pooled BLISS study population, lower proportions of FHS respondents accrued damage over the course of the open-label extension period compared with no FHS respondents (*p* = 0.004; derived from the logrank test; Figure 1). Experience of FHS at the open-label baseline was associated with a lower probability of and/or longer time to subsequent organ damage accrual during the open-label extension period in unadjusted Cox regression analysis [hazard ratio (HR): 0.52; 95% confidence interval (CI): 0.33–0.81; *p* = 0.004; Supplementary Table 1]. This association remained significant after adjustment for potential confounders in multivariable analysis (HR: 0.60; 95% CI: 0.38–0.96; *p* = 0.033; Figure 2 and Supplementary Table 2). However, no associations were seen between experience of FHS at open-label baseline and subsequent organ damage accrual in analysis stratified by SDI organ domain (Supplementary Tables 3, 4).

### 3.2 Level 1 response within each EQ-5D dimension in relation to organ damage accrual

**FIGURE 3:**
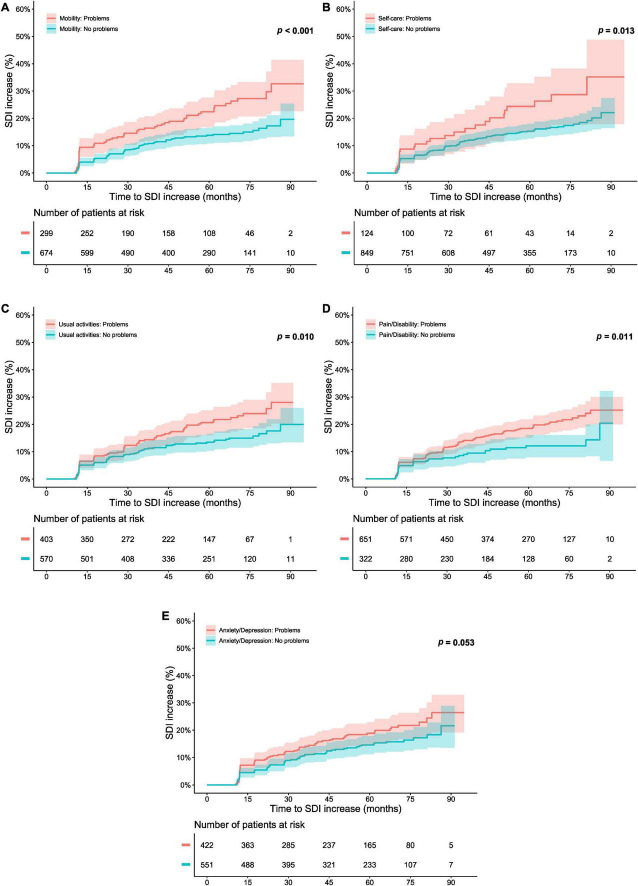
Responses of “no problems” in the separate dimensions of EQ-5D-3L in relation to organ damage accrual. The graphs in the upper panels delineate proportions of patients who accrued organ damage over the course of the open-label study period, stratified into patients who experienced “no problems” with regard to **(A)** mobility, **(B)** self-care, **(C)** usual activities, **(D)** pain/discomfort, and **(E)** anxiety/depression at the open-label baseline, and patients who did not. The lower panel shows numbers of participants in the different groups over time, decreasing due to documentation of SDI increase or data censoring. EQ-5D-3L, three-level version of EQ-5D; FHS, full health state; SDI, Systemic Lupus International Collaborating Clinics (SLICC)/American College of Rheumatology (ACR) Damage Index.

Figure 3 delineates patients reporting “no problems” (severity level 1) and “problems” (severity level 2–3) in each one of the five EQ-5D dimensions at open-label baseline in relation to organ damage accrual throughout the open-label follow-up. A lower proportion of level 1 respondents accrued organ damage during the course of the open-label extension than did other respondents regarding mobility (*p* < 0.001; derived from the logrank test; Figure 3A), self-care (*p* = 0.013; Figure 3B), usual activities (*p* = 0.010; Figure 3C), and pain/discomfort (*p* = 0.011; Figure 3D). Moreover, an association between experience of a level 1 response at open-label baseline and a lower probability of and/or longer time to organ damage accrual was seen with regard to mobility (HR: 0.54; 95% CI: 0.39–0.75; *p* < 0.001), self-care (HR: 0.59; 95% CI: 0.38–0.90; *p* = 0.014), usual activities (HR: 0.66; 95% CI: 0.48–0.91; *p* = 0.011), and pain/discomfort (HR: 0.61; 95% CI: 0.42–0.90; *p* = 0.012; Supplementary Table 1). Of these associations, only the association for mobility remained significant after adjustment for potential confounders in multivariable Cox regression analysis (HR: 0.61; 95% CI: 0.43–0.85; *p* = 0.004; Figure 2 and Supplementary Tables 5–9).

**FIGURE 4:**
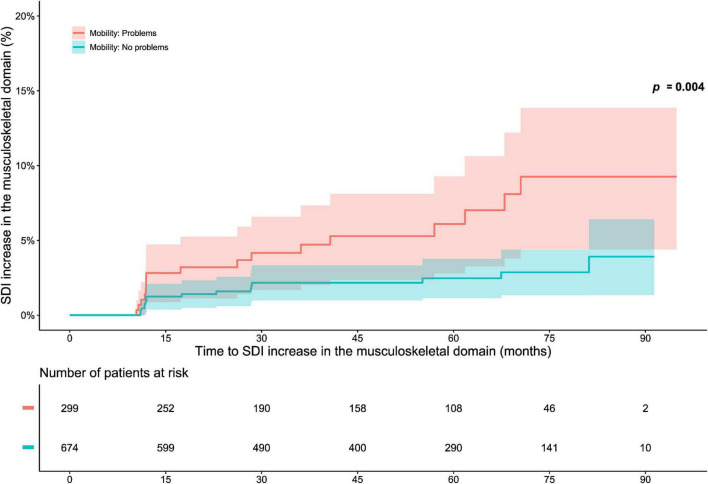
EQ-5D-3L mobility in relation to musculoskeletal damage accrual. The graphs in the upper panel delineate proportions of patients who accrued musculoskeletal damage over the course of the open-label study period, stratified into patients who experienced “no problems” with regard to mobility at the open-label baseline, and patients who did not. The lower panel shows numbers of participants in the two groups over time, decreasing due to documentation of SDI increase or data censoring. EQ-5D-3L, three-level version of EQ-5D; FHS, full health state; SDI, Systemic Lupus International Collaborating Clinics (SLICC)/American College of Rheumatology (ACR) Damage Index.

The analysis of associations between “no problems” within EQ-5D dimensions and organ-specific damage accrual revealed that a lower proportion of level 1 respondents accrued musculoskeletal damage during the course of the open-label phase than did level 2–3 respondents regarding the mobility dimension (*p* = 0.004; derived from the logrank test; Figure 4). Experience of “no problems” regarding mobility was associated with reduced subsequent musculoskeletal damage accrual in time-dependent Cox regression analysis (HR: 0.38; 95% CI: 0.19–0.76; *p* = 0.006). No other associations were documented between “no problems” within the EQ-5D dimensions and organ-specific damage accrual (Supplementary Table 3). Similarly, experience of “no problems” within mobility was negatively correlated with musculoskeletal damage accrual during the open-label follow-up, irrespective of when in time this occurred (φ = −0.08; *p* = 0.008; Supplementary Table 4). We observed no such correlations between “no problems” within the other EQ-5D dimensions and organ-specific damage accrual (Supplementary Table 4).

## 4 Discussion

In the present *post hoc* analysis of data from the BLISS-52 and BLISS-76 open-label extension studies, we found an association between EQ-5D-3L FHS experience at baseline (i.e., post therapeutic intervention with belimumab or placebo on top of non-biological standard therapy) and reduced subsequent organ damage accrual over the course of up to 8 years of open-label follow-up of SLE patients receiving add-on belimumab. Furthermore, experience of “no problems” regarding mobility was associated with a lower hazard to accrue organ damage, particularly musculoskeletal damage.

To the best of the authors’ knowledge, the association between EQ-5D FHS and organ damage accrual has not been studied to date. Several studies have found associations between patient-reported HRQoL and long-term outcomes in people with SLE, but the data have been conflicting (26–29). In cross-sectional studies, there has been a lack of consensus as to whether EQ-5D scores associate with already established organ damage (14–16). Importantly, associations between patient-reported HRQoL experience and SDI increase over time have not been reported in the literature and hence served as the scope of the present work, aiming at an understanding of how SLE patients’ perceptions of health state relate to disease evolution.

Self-reported experience of FHS indicates a high level of wellbeing in all five EQ-5D dimensions. We found that FHS was associated with a reduced hazard to accrue subsequent organ damage, as was “no problems” within the mobility dimension, the latter thus presumably comprising the main driver of the association found for FHS. Interestingly, experience of “no problems” within the mobility dimension of EQ-5D was negatively associated with progression of organ damage in the musculoskeletal SDI domain in particular. The intuitive direction of this association, with unhampered mobility presumably facilitating physical activity and preservation of musculoskeletal function, points to the reliability of the self-reported patient perspective, hence its usefulness in treatment and disease evaluation. Of course, patient-reported outcomes are not meant to substitute but rather be complemental to clinical and laboratory parameters. The findings of this study advocate for their potential usefulness also as a proxy for prognostication.

Several self-assessment instruments of HRQoL exist that have specifically been developed for SLE. Inevitably, the more precise the instrument is, the more time and effort it requires from the respondent to fill in the instrument, which may also result in decreased response frequencies or recruitment of participants in studies, incomplete responses, and incorrect completion (30). For this reason, the briefness of the EQ-5D format paired with its known-group validity (17) and its satisfactory psychometric properties (14) in SLE patients along with its ability to predict long-term disease outcome as shown in the present study suggest that EQ-5D is a feasible and clinically relevant HRQoL instrument to use in SLE practices and studies.

During the past few decades, treatment of common chronic illnesses such as hypertension and diabetes has evolved from symptomatic to target-based (31). In the wake of this trend, the principle of treat-to-target has evolved during the past decade as a strategy for the management of rheumatic diseases e.g., rheumatoid arthritis (RA) (32). In later years, it has also been successfully applied in SLE, where remission (33, 34) and Lupus Low Disease Activity State (LLDAS) (35, 36) have been demonstrated to associate with deceleration of organ damage accrual (37) and favourable HRQoL experience (38, 39), and hence receive increasing endorsement as treatment targets (31). However, in contrast to the Disease Activity Score (DAS)-28-based definition of remission used in RA (40), which encompasses patient-reported global health, common definitions of remission and low disease activity in SLE do not include patient-reported components. While consensus upon how patient-reported outcomes should be integrated in SLE to serve as outcome measures used in studies and clinical practice has yet to be achieved (10), our findings advocate that patient-reported HRQoL by means of EQ-5D holds promise as a complemental component to current definitions. However, it is important to note that addition of patient-reported components to already validated outcome measures is a highly speculative notion until their added value has been thoroughly investigated. Nevertheless, the need of adequately capturing the patient perspective is imperative, as highlighted by the apparent discrepancies between patients’ and clinicians’ priorities (9) and perceptions of health state (41–43). Along the same lines, a recent study showed that substantial proportions of patients who had attained adequate responses to treatment still experienced poor HRQoL (7). Altogether, the prospect of including patient-reported components in definitions of response to therapy, remission, or low disease activity may form an essential constituent of the treat-to-target research agenda, with the present investigation lending support for EQ-5D as a useful tool for capturing SLE patients’ HRQoL experience.

Limitations included the *post hoc* nature of our study design, the lack of EQ-5D-3L data during the open-label phase, as well as the lack of adjustments for potentially confounding comorbidities, such as fibromyalgia. Another important limitation was the different follow-up times of 52 and 76 weeks in the BLISS-52 and BLISS-76 clinical trials, respectively, which may have favoured attainability of “no problems” in the different EQ-5D dimensions or FHS in BLISS-76 where the patients were followed for approximately 50% longer time than in BLISS-52 before entering the open-label phase. However, a previous analysis from our group showed that proportions of EQ-5D-3L FHS responses plateaued from week 52 through week 76 in BLISS-76 (17). Furthermore, the selected population of the trials consisting mainly of articular and/or mucocutaneous SLE and excluding severe active renal and CNS lupus may not be considered fully representative of real-life clinical settings. It is also worth noting that relatively few patients accrued organ damage during the open-label extension studies, as indicated herein as well as by the work of others (20). Particular strengths of the study included the large and ethnically diverse SLE population of the BLISS trials, and the follow-up period of up to 8 years.

In this study, patient-reported experience of EQ-5D-3L full health state and “no problems” in the mobility dimension after therapeutic intervention heralded a reduced hazard to accrue subsequent organ damage, especially in the musculoskeletal domain. In the era of treat-to-target strategies, our findings canvass the use of EQ-5D in a discretised manner as a clinically relevant treatment target in SLE, captured by the patients themselves, complemental to clinical and laboratory parameters. Presumably, more favourable long-term outcomes may be achieved for SLE patients if we target towards the best possible patient-reported HRQoL experience, along with the lowest possible disease activity and, when possible, remission.

### 4.1 Patient involvement

Patient research partners were involved in the study concept and design, interpretation of data, and editing of the manuscript.

## Data availability statement

The raw data supporting the conclusions of this article will be made available by the authors, without undue reservation.

## Ethics statement

The BLISS study protocols were reviewed and approved by regional Ethics Review Boards for all participating centres, and the study protocol for this *post-hoc* analysis was reviewed and approved by the Swedish Ethical Review Authority (2019-05498). Written informed consent was obtained from all study participants prior to enrolment in BLISS-52 and BLISS-76 and their OL extension phases.

## Author contributions

JL, SZ, YE, DG, AG, and IP: conception and design of the work. JL, SZ, SE, DG, AG, and IP: data management. JL, SZ, GP, YE, EH, MR, DG, AG, and IP: statistical analysis and interpretation of data. GP and YE: patient research partners. JL, SZ, SE, AB, GP, YE, EH, MR, DG, AG, and IP: critical revision of the manuscript for important intellectual content. All authors reviewed and approved the final version of the manuscript prior to submission and agreed to be accountable for all aspects of the work.
