# Supplementary material for: EQ-5D full health state after therapy heralds reduced hazard to accrue subsequent organ damage in systemic lupus erythematosus
Source: Front Med (Lausanne). 2022 Dec 20;9:1092325. doi: 10.3389/fmed.2022.1092325 (PMC9807754; doi:10.3389/fmed.2022.1092325)
Supplement: Supplementary file 1 [file Data_Sheet_1.PDF]

Supplementary Material

Table of Contents

1.1 Supplementary Table S1. Associations between EQ-5D-3L responses and organ damage accrual....2

1.2 Supplementary Table S2. Associations between EQ-5D-3L responses and organ damage accrual....3

1.3 Supplementary Table S3. Associations between EQ-5D-3L responses and organ damage accrual....4

1.4 Supplemental Table S4. Phi (φ) correlations between EQ-5D-3L responses and organ damage accrual....6

1.5 Supplementary Table S5. Associations between EQ-5D-3L responses and organ damage accrual....8

1.6 Supplementary Table S6. Associations between EQ-5D-3L responses and organ damage accrual....9

1.7 Supplementary Table S7. Associations between EQ-5D-3L responses and organ damage accrual....10

1.8 Supplementary Table S8. Associations between EQ-5D-3L responses and organ damage accrual....11

1.9 Supplementary Table S9. Associations between EQ-5D-3L responses and organ damage accrual....12

1.1 Supplementary Table S1. Associations between EQ-5D-3L responses and organ damage accrual

	Coefficient	HR	95% CI	*p* value
EQ-5D-3L FHS at baseline	-0.66	0.52	0.33−0.81	**0.004**
EQ-5D-3L dimensions at baseline (no problems)				
Mobility	-0.61	0.54	0.39−0.75	**<0.001**
Self-care	-0.53	0.59	0.38−0.90	**0.014**
Usual activities	-0.42	0.66	0.48−0.91	**0.011**
Pain/discomfort	-0.49	0.61	0.42−0.90	**0.012**
Anxiety/depression	-0.32	0.73	0.53−1.01	0.054

Results from univariable proportional hazards (Cox) regression analysis, with SDI increase during the open-label extension BLISS study follow-up as the dependent variable. The total number of patients with available data was 973. Data are presented as the coefficients, hazard ratios (HR), 95% confidence intervals (CI), and *p* values. Statistically significant *p* values are in bold. CI: confidence interval; EQ-5D-3L: three-level version of EQ-5D; FHS: full health state; HR: hazard ratio; SDI: Systemic Lupus International Collaborating Clinics (SLICC)/American College of Rheumatology (ACR) Damage Index.

1.2 Supplementary Table S2. Associations between EQ-5D-3L responses and organ damage accrual

	**Coefficient**	**HR**	**95% CI**	***p* value**
EQ-5D FHS at baseline	-0.50	0.60	0.38–0.96	**0.033**
Age at baseline (years)	0.02	1.02	1.00–1.04	**0.021**
Female sex	0.18	1.20	0.58–2.48	0.621
Ethnicity (reference: White/Caucasian)				
Asian	0.18	1.20	0.76–1.90	0.437
Black/African American	0.62	1.86	1.14–3.05	**0.013**
Indigenous American*	-0.31	0.74	0.46–1.18	0.203
SDI score at baseline	0.41	1.51	1.06–2.15	**0.021**
Antimalarial agents at week 52†	-0.01	0.99	0.70–1.40	0.945
Mean prednisone equivalent dose during follow-up (mg)	0.01	1.01	0.99–1.03	0.571
Immunosuppressants at week 52				
Azathioprine	0.28	1.32	0.89–1.97	0.172
Methotrexate	0.22	1.25	0.77–2.03	0.363
Mycophenolic acid	0.05	1.05	0.59–1.88	0.858
Other immunosuppressants‡	-0.18	0.84	0.20–3.47	0.805

Results from multivariable proportional hazards (Cox) regression analysis, with SDI increase during the open-label extension BLISS study follow-up as the dependent variable. The total number of patients with available data was 972. Data are presented as the coefficients, hazard ratios (HR), 95% confidence intervals (CI), and *p* values. Statistically significant *p* values are in bold. CI: confidence interval; EQ-5D-3L: three-level version of EQ-5D; FHS: full health state; HR: hazard ratio; SDI: Systemic Lupus International Collaborating Clinics (SLICC)/American College of Rheumatology (ACR) Damage Index.

* Alaska Native or American Indian from North, South or Central America.

† Hydroxychloroquine, chloroquine, mepacrine, mepacrine hydrochloride or quinine sulfate.

‡ Cyclosporine, oral cyclophosphamide, mizoribine or thalidomide.

1.3 Supplementary Table S3. Associations between EQ-5D-3L responses and organ damage accrual

SDI items	n	Coefficient	HR	95% CI	*p* value
**Ocular**					
EQ-5D-3L FHS at baseline	27	-0.68	0.51	0.18−1.47	0.212
EQ-5D-3L dimensions at baseline (no problems)					
Mobility	27	-0.68	0.51	0.24−1.08	0.080
Self-care	27	-0.59	0.56	0.21−1.47	0.238
Usual activities	27	-0.05	0.95	0.44−2.05	0.890
Pain/discomfort	27	-0.16	0.85	0.37−1.94	0.701
Anxiety/depression	27	-0.38	0.69	0.32−1.46	0.330
**Neuropsychiatric**					
EQ-5D-3L FHS at baseline	15	-0.79	0.45	0.10−2.01	0.299
EQ-5D-3L dimensions at baseline (no problems)					
Mobility	15	-0.74	0.48	0.17−1.32	0.153
Self-care	15	-1.03	0.36	0.11−1.13	0.079
Usual activities	15	-0.51	0.60	0.22−1.66	0.324
Pain/discomfort	15	-0.68	0.51	0.14−1.80	0.294
Anxiety/depression	15	-0.17	0.85	0.31−2.34	0.748
**Renal**					
EQ-5D-3L FHS at baseline	8	-0.87	0.42	0.05−3.41	0.417
EQ-5D-3L dimensions at baseline (no problems)					
Mobility	8	-0.38	0.68	0.16−2.87	0.604
Self-care	8	-0.11	0.89	0.11−7.26	0.915
Usual activities	8	-0.38	0.68	0.17−2.72	0.586
Pain/discomfort	8	-0.40	0.67	0.14−3.33	0.626
Anxiety/depression	8	-1.41	0.24	0.05−1.21	0.085
**Pulmonary**					
EQ-5D-3L FHS at baseline	4	-0.02	0.98	0.10−9.38	0.983
EQ-5D-3L dimensions at baseline (no problems)					
Mobility	4	18.55	113541632	0−+inf	0.998
Self-care	4	18.18	78956599	0−+inf	0.999
Usual activities	4	19.86	419926995	0−+inf	0.999
Pain/discomfort	4	-0.41	0.67	0.07−6.41	0.725
Anxiety/depression	4	19.92	449784613	0−+inf	0.999
**Cardiovascular**					
EQ-5D-3L FHS at baseline	10	-18.48	0.00	0−+inf	0.997
EQ-5D-3L dimensions at baseline (no problems)					
Mobility	10	-0.05	0.95	0.24−3.66	0.937
Self-care	10	-0.68	0.51	0.11−2.38	0.389
Usual activities	10	-1.23	0.29	0.08−1.13	0.074
Pain/discomfort	10	-1.49	0.23	0.03−1.78	0.158
Anxiety/depression	10	-1.17	0.31	0.08−1.21	0.091
**Peripheral vascular**					
EQ-5D-3L FHS at baseline	6	-18.47	0.00	0−+inf	0.998
EQ-5D-3L dimensions at baseline (no problems)					
Mobility	6	-0.91	0.40	0.08−2.00	0.267
Self-care	6	18.19	79107898	0−+inf	0.999
Usual activities	6	-1.11	0.33	0.06−1.80	0.200
Pain/discomfort	6	-0.87	0.42	0.05−3.59	0.427
Anxiety/depression	6	0.39	1.48	0.27−8.10	0.649
**Gastrointestinal**					
EQ-5D-3L FHS at baseline	8	-0.86	0.42	0.05−3.45	0.422
EQ-5D-3L dimensions at baseline (no problems)					

Mobility	8	-0.89	0.41	0.10−1.64	0.206
Self-care	8	18.19	79576154	0−+inf	0.998
Usual activities	8	-0.91	0.40	0.10−1.68	0.212
Pain/discomfort	8	-1.22	0.29	0.04−2.39	0.252
Anxiety/depression	8	0.21	1.23	0.29−5.14	0.778
**Musculoskeletal**					
EQ-5D-3L FHS at baseline	33	0.10	1.10	0.51−2.37	0.806
EQ-5D-3L dimensions at baseline (no problems)					
Mobility	33	-0.96	0.38	0.19−0.76	**0.006**
Self-care	33	-0.75	0.47	0.21−1.09	0.080
Usual activities	33	-0.46	0.63	0.32−1.25	0.186
Pain/discomfort	33	-0.27	0.76	0.35−1.64	0.489
Anxiety/depression	33	0.01	1.01	0.50−2.01	0.989
**Skin**					
EQ-5D-3L FHS at baseline	10	-1.12	0.33	0.04−2.59	0.290
EQ-5D-3L dimensions at baseline (no problems)					
Mobility	10	-0.49	0.62	0.17−2.18	0.452
Self-care	10	0.13	1.14	0.14−9.01	0.900
Usual activities	10	0.46	1.59	0.41−6.14	0.503
Pain/discomfort	10	-0.14	0.87	0.22−3.36	0.839
Anxiety/depression	10	-0.71	0.49	0.14−1.74	0.269
**Premature gonadal failure**					
EQ-5D-3L FHS at baseline	3	-18.48	0.00	0−+inf	0.999
EQ-5D-3L dimensions at baseline (no problems)					
Mobility	3	-1.61	0.20	0.02−2.21	0.190
Self-care	3	-1.41	0.24	0.02−2.70	0.251
Usual activities	3	-1.08	0.34	0.03−3.73	0.376
Pain/discomfort	3	-19.67	0.00	0−+inf	0.999
Anxiety/depression	3	-1.01	0.37	0.03−4.03	0.411
**Diabetes**					
EQ-5D-3L FHS at baseline	12	-1.33	0.26	0.03−2.04	0.202
EQ-5D-3L dimensions at baseline (no problems)					
Mobility	12	-0.94	0.39	0.13−1.22	0.106
Self-care	12	-1.00	0.37	0.10−1.36	0.134
Usual activities	12	-0.78	0.46	0.15−1.45	0.184
Pain/discomfort	12	-0.90	0.41	0.09−1.86	0.245
Anxiety/depression	12	-0.65	0.52	0.17−1.65	0.268
**Malignancy**					
EQ-5D-3L FHS at baseline	11	-0.43	0.65	0.14−3.01	0.581
EQ-5D-3L dimensions at baseline (no problems)					
Mobility	11	0.58	1.79	0.39−8.30	0.456
Self-care	11	-0.61	0.54	0.12−2.52	0.436
Usual activities	11	-0.60	0.55	0.17−1.80	0.320
Pain/discomfort	11	-0.29	0.75	0.20−2.82	0.668
Anxiety/depression	11	-0.48	0.62	0.19−2.02	0.424

Results from univariable proportional hazards (Cox) regression analysis, with SDI increase within different organ domains during the open-label extension BLISS study follow-up as the dependent variable. The number of patients with organ damage accrual within each organ domain is indicated by n. The total number of patients with available data was 973. Data are presented as the coefficients, hazard ratios (HR), 95% confidence intervals (CI), and *p* values. Statistically significant *p* values are in bold. CI: confidence interval; EQ-5D-3L: three-level version of EQ-5D; FHS: full health state; inf: infinite; HR: hazard ratio; SDI: Systemic Lupus International Collaborating Clinics (SLICC)/American College of Rheumatology (ACR) Damage Index.

1.4 Supplemental Table S4. Phi (φ) correlations between EQ-5D-3L responses and organ damage accrual

	n	φ	*p* value
**Ocular**			
EQ-5D-3L FHS at baseline	27	-0.04	0.221
EQ-5D-3L dimensions at baseline (no problems)			
Mobility	27	-0.05	0.117
Self-care	27	-0.03	0.362
Usual activities	27	0.00	0.942
Pain/discomfort	27	-0.01	0.698
Anxiety/depression	27	-0.03	0.368
**Neuropsychiatric**			
EQ-5D-3L FHS at baseline	15	-0.03	0.298
EQ-5D-3L dimensions at baseline (no problems)			
Mobility	15	-0.04	0.178
Self-care	15	-0.05	0.103
Usual activities	15	-0.03	0.346
Pain/discomfort	15	-0.03	0.278
Anxiety/depression	15	-0.01	0.795
**Renal**			
EQ-5D-3L FHS at baseline	8	-0.03	0.417
EQ-5D-3L dimensions at baseline (no problems)			
Mobility	8	-0.01	0.677
Self-care	8	0.00	0.983
Usual activities	8	-0.02	0.621
Pain/discomfort	8	-0.02	0.626
Anxiety/depression	8	-0.06	0.070
**Pulmonary**			
EQ-5D-3L FHS at baseline	4	0.00	0.995
EQ-5D-3L dimensions at baseline (no problems)			
Mobility	4	0.04	0.182
Self-care	4	0.02	0.444
Usual activities	4	0.05	0.092
Pain/discomfort	4	-0.01	0.731
Anxiety/depression	4	0.06	0.080
**Cardiovascular**			
EQ-5D-3L FHS at baseline	10	-0.06	0.068
EQ-5D-3L dimensions at baseline (no problems)			
Mobility	10	0.00	0.960
Self-care	10	-0.02	0.490
Usual activities	10	-0.06	0.065
Pain/discomfort	10	-0.05	0.119
Anxiety/depression	10	-0.05	0.088
**Peripheral vascular**			
EQ-5D-3L FHS at baseline	6	-0.05	0.158
EQ-5D-3L dimensions at baseline (no problems)			
Mobility	6	-0.03	0.305
Self-care	6	0.03	0.348
Usual activities	6	-0.04	0.208
Pain/discomfort	6	-0.03	0.392
Anxiety/depression	6	0.02	0.619
**Gastrointestinal**			
EQ-5D-3L FHS at baseline	8	-0.03	0.417
EQ-5D-3L dimensions at baseline (no problems)			
Mobility	8	-0.04	0.236

Self-care	8	0.03	0.278
Usual activities	8	-0.04	0.225
Pain/discomfort	8	-0.04	0.214
Anxiety/depression	8	0.01	0.737
**Musculoskeletal**			
EQ-5D-3L FHS at baseline	33	0.01	0.746
EQ-5D-3L dimensions at baseline (no problems)			
Mobility	33	-0.08	**0.008**
Self-care	33	-0.05	0.138
Usual activities	33	-0.04	0.231
Pain/discomfort	33	-0.02	0.470
Anxiety/depression	33	0.00	0.911
**Skin**			
EQ-5D-3L FHS at baseline	10	-0.04	0.275
EQ-5D-3L dimensions at baseline (no problems)			
Mobility	10	-0.02	0.524
Self-care	10	0.01	0.794
Usual activities	10	0.02	0.462
Pain/discomfort	10	-0.01	0.835
Anxiety/depression	10	-0.03	0.287
**Premature gonadal failure**			
EQ-5D-3L FHS at baseline	3	-0.03	0.319
EQ-5D-3L dimensions at baseline (no problems)			
Mobility	3	-0.04	0.177
Self-care	3	-0.03	0.285
Usual activities	3	-0.03	0.374
Pain/discomfort	3	-0.04	0.223
Anxiety/depression	3	-0.03	0.415
**Diabetes**			
EQ-5D-3L FHS at baseline	12	-0.04	0.183
EQ-5D-3L dimensions at baseline (no problems)			
Mobility	12	-0.05	0.146
Self-care	12	-0.04	0.201
Usual activities	12	-0.04	0.232
Pain/discomfort	12	-0.04	0.224
Anxiety/depression	12	-0.03	0.293
**Malignancy**			
EQ-5D-3L FHS at baseline	11	-0.02	0.606
EQ-5D-3L dimensions at baseline (no problems)			
Mobility	11	0.03	0.365
Self-care	11	-0.02	0.587
Usual activities	11	-0.03	0.375
Pain/discomfort	11	-0.01	0.680
Anxiety/depression	11	-0.02	0.453

Phi (φ) correlations between EQ-5D-3L responses and SDI increase within different organ domains during the open-label extension BLISS study follow-up. The number of patients with organ damage accrual within each organ domain is indicated by n. The total number of patients with available data was 973. Statistically significant *p* values are in bold. EQ-5D-3L: three-level version of EQ-5D; FHS: full health state; SDI: Systemic Lupus International Collaborating Clinics (SLICC)/American College of Rheumatology (ACR) Damage Index.

1.5 Supplementary Table S5. Associations between EQ-5D-3L responses and organ damage accrual

	Coefficient	HR	95% CI	*p* value
EQ-5D mobility at baseline (no problems)	-0.50	0.61	0.43–0.85	**0.004**
Age at baseline (years)	0.02	1.02	1.00–1.04	**0.023**
Female sex	0.25	1.29	0.62–2.64	0.496
Ethnicity (reference: White/Caucasian)				
Asian	0.20	1.22	0.77–1.92	0.404
Black/African American	0.64	1.91	1.17–3.11	**0.010**
Indigenous American*	-0.29	0.75	0.47–1.21	0.237
SDI score at baseline	0.43	1.54	1.08–2.18	**0.016**
Antimalarial agents at week 52†	0.03	1.03	0.72–1.45	0.886
Mean prednisone equivalent dose during follow-up (mg)	0.01	1.01	0.98–1.03	0.639
Immunosuppressants at week 52				
Azathioprine	0.28	1.32	0.89–1.97	0.170
Methotrexate	0.14	1.15	0.71–1.87	0.579
Mycophenolic acid	0.08	1.08	0.61–1.93	0.789
Other immunosuppressants‡	-0.16	0.86	0.21–3.55	0.830

Results from multivariable proportional hazards (Cox) regression analysis, with SDI increase during the open-label extension BLISS study follow-up as the dependent variable. The total number of patients with available data was 972. Data are presented as the coefficients, hazard ratios (HR), 95% confidence intervals (CI), and *p* values. Statistically significant *p* values are in bold. CI: confidence interval; EQ-5D-3L: three-level version of EQ-5D; HR: hazard ratio; SDI: Systemic Lupus International Collaborating Clinics (SLICC)/American College of Rheumatology (ACR) Damage Index.

* Alaska Native or American Indian from North, South or Central America.

† Hydroxychloroquine, chloroquine, mepacrine, mepacrine hydrochloride or quinine sulfate.

‡ Cyclosporine, oral cyclophosphamide, mizoribine or thalidomide.

1.6 Supplementary Table S6. Associations between EQ-5D-3L responses and organ damage accrual

	**Coefficient**	**HR**	**95% CI**	***p* value**
EQ-5D self-care at baseline (no problems)	-0.40	0.67	0.44–1.03	0.069
Age at baseline (years)	0.02	1.02	1.00–1.04	**0.011**
Female sex	0.27	1.32	0.64–2.71	0.457
Ethnicity (reference: White/Caucasian)				
Asian	0.17	1.18	0.75–1.87	0.472
Black/African American	0.61	1.84	1.12–3.00	**0.016**
Indigenous American*	-0.32	0.73	0.45–1.17	0.187
SDI score at baseline	0.43	1.54	1.08–2.18	**0.016**
Antimalarial agents at week 52†	0.00	1.00	0.71–1.41	0.994
Mean prednisone equivalent dose during follow-up (mg)	0.01	1.01	0.98–1.03	0.595
Immunosuppressants at week 52				
Azathioprine	0.25	1.29	0.87–1.92	0.211
Methotrexate	0.24	1.27	0.78–2.01	0.334
Mycophenolic acid	0.02	1.02	0.57–1.81	0.957
Other immunosuppressants‡	-0.12	0.89	0.21–3.68	0.867

Results from multivariable proportional hazards (Cox) regression analysis, with SDI increase during the open-label extension BLISS study follow-up as the dependent variable. The total number of patients with available data was 972. Data are presented as the coefficients, hazard ratios (HR), 95% confidence intervals (CI), and *p* values. Statistically significant *p* values are in bold. CI: confidence interval; EQ-5D-3L: three-level version of EQ-5D; HR: hazard ratio; SDI: Systemic Lupus International Collaborating Clinics (SLICC)/American College of Rheumatology (ACR) Damage Index.

* Alaska Native or American Indian from North, South or Central America.

† Hydroxychloroquine, chloroquine, mepacrine, mepacrine hydrochloride or quinine sulfate.

‡ Cyclosporine, oral cyclophosphamide, mizoribine or thalidomide.

1.7 Supplementary Table S7. Associations between EQ-5D-3L responses and organ damage accrual

	Coefficient	HR	95% CI	*p* value
EQ-5D usual activities at baseline (no problems)	-0.32	0.73	0.52–1.02	0.064
Age at baseline (years)	0.02	1.02	1.00–1.04	**0.017**
Female sex	0.22	1.24	0.60–2.55	0.558
Ethnicity (reference: White/Caucasian)				
Asian	0.21	1.24	0.78–1.97	0.368
Black/African American	0.64	1.89	1.16–3.09	**0.011**
Indigenous American*	-0.29	0.75	0.46–1.20	0.232
SDI score at baseline	0.44	1.56	1.10–2.21	**0.014**
Antimalarial agents at week 52†	-0.01	0.99	0.70–1.39	0.940
Mean prednisone equivalent dose during follow-up (mg)	0.01	1.01	0.98–1.03	0.589
Immunosuppressants at week 52				
Azathioprine	0.26	1.30	0.87–1.94	0.195
Methotrexate	0.19	1.21	0.75–1.97	0.433
Mycophenolic acid	0.02	1.02	0.57–1.82	0.937
Other immunosuppressants‡	-0.15	0.86	0.21–3.56	0.833

Results from multivariable proportional hazards (Cox) regression analysis, with SDI increase during the open-label extension BLISS study follow-up as the dependent variable. The total number of patients with available data was 972. Data are presented as the coefficients, hazard ratios (HR), 95% confidence intervals (CI), and *p* values. Statistically significant *p* values are in bold. CI: confidence interval; EQ-5D-3L: three-level version of EQ-5D; HR: hazard ratio; SDI: Systemic Lupus International Collaborating Clinics (SLICC)/American College of Rheumatology (ACR) Damage Index.

* Alaska Native or American Indian from North, South or Central America.
† Hydroxychloroquine, chloroquine, mepacrine, mepacrine hydrochloride or quinine sulfate.
‡ Cyclosporine, oral cyclophosphamide, mizoribine or thalidomide.

1.8 Supplementary Table S8. Associations between EQ-5D-3L responses and organ damage accrual.

	Coefficient	HR	95% CI	*p* value
EQ-5D pain/discomfort at baseline (no problems)	-0.37	0.69	0.47–1.02	0.063
Age at baseline (years)	0.02	1.02	1.00–1.04	**0.018**
Female sex	0.21	1.24	0.60–2.55	0.565
Ethnicity (reference: White/Caucasian)				
Asian	0.18	1.20	0.76–1.90	0.433
Black/African American	0.62	1.86	1.14–3.05	**0.013**
Indigenous American*	-0.32	0.73	0.45–1.17	0.189
SDI score at baseline	0.43	1.53	1.08–2.18	**0.017**
Antimalarial agents at week 52†	-0.01	0.99	0.70–1.39	0.937
Mean prednisone equivalent dose during follow-up (mg)	0.01	1.01	0.99–1.03	0.544
Immunosuppressants at week 52				
Azathioprine	0.27	1.31	0.88–1.95	0.189
Methotrexate	0.22	1.24	0.77–2.02	0.375
Mycophenolic acid	0.06	1.06	0.59–1.89	0.844
Other immunosuppressants‡	-0.18	0.84	0.20–3.47	0.807

Results from multivariable proportional hazards (Cox) regression analysis, with SDI increase during the open-label extension BLISS study follow-up as the dependent variable. The total number of patients with available data was 972. Data are presented as the coefficients, hazard ratios (HR), 95% confidence intervals (CI), and *p* values. Statistically significant *p* values are in bold. CI: confidence interval; EQ-5D-3L: three-level version of EQ-5D; HR: hazard ratio; SDI: Systemic Lupus International Collaborating Clinics (SLICC)/American College of Rheumatology (ACR) Damage Index.

* Alaska Native or American Indian from North, South or Central America.
† Hydroxychloroquine, chloroquine, mepacrine, mepacrine hydrochloride or quinine sulfate.
‡ Cyclosporine, oral cyclophosphamide, mizoribine or thalidomide.

1.9 Supplementary Table S9. Associations between EQ-5D-3L responses and organ damage accrual

	Coefficient	HR	95% CI	*p* value
EQ-5D anxiety/depression at baseline (no problems)	-0.27	0.76	0.55–1.06	0.105
Age at baseline (years)	0.02	1.02	1.00–1.04	**0.011**
Female sex	0.21	1.24	0.60–2.55	0.564
Ethnicity (reference: White/Caucasian)				
Asian	0.15	1.17	0.74–1.84	0.507
Black/African American	0.65	1.92	1.17–3.14	**0.010**
Indigenous American*	-0.33	0.72	0.45–1.16	0.176
SDI score at baseline	0.43	1.53	1.08–2.18	**0.017**
Antimalarial agents at week 52†	-0.01	0.99	0.70–1.39	0.939
Mean prednisone equivalent dose during follow-up (mg)	0.00	1.00	0.98–1.03	0.652
Immunosuppressants at week 52				
Azathioprine	0.26	1.30	0.87–1.94	0.197
Methotrexate	0.23	1.26	0.78–2.04	0.347
Mycophenolic acid	0.03	1.03	0.58–1.84	0.918
Other immunosuppressants‡	-0.20	0.82	0.20–3.41	0.786

Results from multivariable proportional hazards (Cox) regression analysis, with SDI increase during the open-label extension BLISS study follow-up as the dependent variable. The total number of patients with available data was 972. Data are presented as the coefficients, hazard ratios (HR), 95% confidence intervals (CI), and *p* values. Statistically significant *p* values are in bold. CI: confidence interval; EQ-5D-3L: three-level version of EQ-5D; HR: hazard ratio; SDI: Systemic Lupus International Collaborating Clinics (SLICC)/American College of Rheumatology (ACR) Damage Index.

* Alaska Native or American Indian from North, South or Central America.

† Hydroxychloroquine, chloroquine, mepacrine, mepacrine hydrochloride or quinine sulfate.

‡ Cyclosporine, oral cyclophosphamide, mizoribine or thalidomide.
